# High acceptability of a contraceptive vaginal ring among women in Kigali, Rwanda

**DOI:** 10.1371/journal.pone.0199096

**Published:** 2018-06-18

**Authors:** Evelyne Kestelyn, Jennifer Ilo Van Nuil, Marie Michelle Umulisa, Grace Umutoni, Alice Uwingabire, Lambert Mwambarangwe, Mireille Uwineza, Stephen Agaba, Tania Crucitti, Janneke van de Wijgert, Thérèse Delvaux, for the Ring Plus Study Group

**Affiliations:** 1 Rinda Ubuzima, Kigali, Rwanda; 2 University of Liverpool, Institute of Infection and Global Health, Liverpool, United kingdom; 3 Institute of Tropical Medicine, Antwerp, Belgium; Massachusetts General Hospital, UNITED STATES

## Abstract

**Background:**

Introduction of contraceptive vaginal rings (CVRs) could expand the contraceptive method mix reducing the unmet need for family planning in Rwanda, but data on acceptability of CVRs from low and middle-income countries are lacking.

**Methods:**

This study explores acceptability of contraceptive vaginal ring (NuvaRing) use in Kigali, Rwanda using a mixed methods approach. We collected quantitative and qualitative data before, during and after conducting a clinical trial, using Case Report Forms, Interviewer Administered Questionnaires, In Depth Interviews and Focus Group Discussions. We analyzed the data using an existing theoretical framework including product attributes, relationship attributes and sexual encounter attributes as well as the contextual environment.

**Results:**

Our data showed that initial worries reduced over time with actual ring use and ring insertions and removals were described as easy. Most women did not feel the ring during daily activities, appreciated the lack of perceived negative side effects and the increased lubrication. Relationship attributes and sexual encounter attributes such as sexual comfort played a significant role in ring acceptability of the participants and their partners. The contextual environment including Rwandan cultural norms around sexuality positively influenced the acceptance of the NuvaRing. Overall satisfaction was high.

**Conclusions:**

Acceptability of the Nuvaring was high among study participants and represents a promising option that could contribute to lowering the unmet need for family planning in Rwanda.

## Introduction

Provision of family planning services remains inadequate in most African countries with an unmet need for contraception resulting in unwanted pregnancies associated with poor maternal and child health outcomes. Although Rwanda has made significant progress in improving maternal and child health, including increasing the availability and coverage of family planning, more can be achieved [1–3]. Currently 48% of married women and 35% of unmarried sexually active women in Rwanda are using a modern contraceptive method [4]. Contraceptive use prevalence is similar across the regions, and in the urban and rural female populations, with an overall national unmet need of 19% [4]. Expansion of the contraceptive options or method mix might reduce this gap.

In recent years, contraceptive vaginal rings (CVRs) have become popular in the countries where they are available. Two CVRs have been marketed in select countries to date: the etonogestrel ethinyl estradiol Nuvaring and the progesterone only Progering® for breastfeeding women [5]. A one-year nestorone ethinyl estradiol ring is currently in the final stages of registration [6]. Numerous studies have documented the safety, tolerability and efficacy of CVRs, as well as several benefits including menstrual cycle control, ease of use, and being user-controlled [7–13]. Nuvaring clinical trials in Europe and North America have shown good acceptability [14–19], but data from low and middle income countries are limited [20]. Some CVR acceptability studies were conducted in Latin America in the early eighties and showed good acceptability [21–22]. Recent studies in women of reproductive age in India, and in breastfeeding women in Kenya, Nigeria, and Senegal, have also shown good acceptability [23–26].

Research on vaginal rings regained momentum with the development of vaginal microbicides for HIV prevention and multipurpose prevention tools (for prevention of pregnancy, HIV, and other sexually transmitted infections (STIs)) [27–29]. In contrast to CVR research, most studies on HIV preventive or multipurpose vaginal rings acceptability and adherence to date have been conducted in Africa [30–33]. This research highlighted the importance of assessing health and sexual behaviors in depth in order to improve our understanding of vaginal ring acceptability and adherence, especially since vaginal rings might interfere with sexual relationships [34]. The objective of our study is to explore acceptability of CVR (NuvaRing) use in Rwanda using both quantitative and qualitative methods.

## Methods

### Study design and setting

This acceptability study was a component of a clinical trial we conducted from June 2013 to March 2014 at the Rinda Ubuzima research site located in Kigali, the urban capital city of Rwanda (NCT01796613). This open label single-centre randomized controlled trial of intermittent versus continuous use of a contraceptive vaginal ring aimed to evaluate NuvaRing safety on the vaginal environment but also included an extensive social science component using a mixed methods approach (quantitative and qualitative data collection) to assess NuvaRing feasibility, acceptability, and adherence [35].

The Rwandan National Ethics Committee (approval number 481/RNEC/2013) and the ethics committees of the Institute of Tropical Medicine (ITM) in Antwerp, Belgium (approval number 864/13), the University Teaching Hospital in Antwerp, Belgium (approval number 13/7/85) and the University of Liverpool in Liverpool, UK (approval number RETG000639IREC) approved the study.

### Study population

Study participants included women between 18 and 35 years old, willing to provide informed consent, HIV negative, sexually active, and in good physical and mental health. They were not currently using a modern contraceptive method but were interested in and eligible for NuvaRing® use [35].

### Study recruitment and procedures

**Fig 1:**
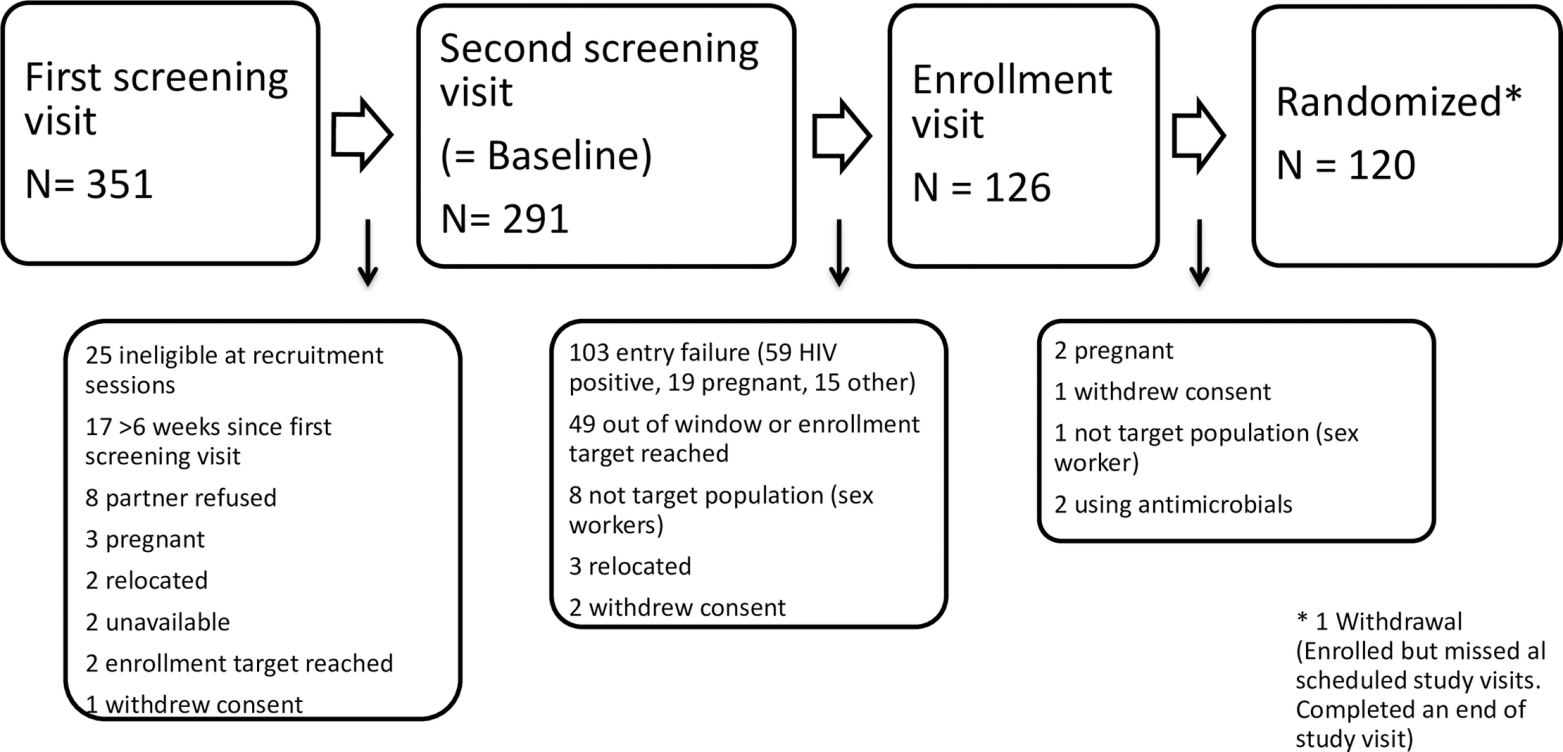
Flow chart of the study process detailing screen failure rates. The study screen flow is presented detailing screen failure rates.

Community mobilizers recruited potential participants at established recruitment sites from previous HIV prevention trials and were, as a result, at above-average risk for HIV/STIs and unplanned pregnancies. The study team screened the women in the study clinic and assessed their eligibility based on the criteria indicated above. Before enrolment, we asked all women whether they would be willing to participate in in depth interviews (IDIs) and/or focus group discussions (FGDs) prior, during and/or after the clinical trial [Fig 1]. At all ring removal follow-up visits, a clinician conducted a physical and pelvic exam, completed the clinic case report forms (CRF), and an interviewer-administered questionnaire (IAQ).

### Data collection

**Fig 2:**
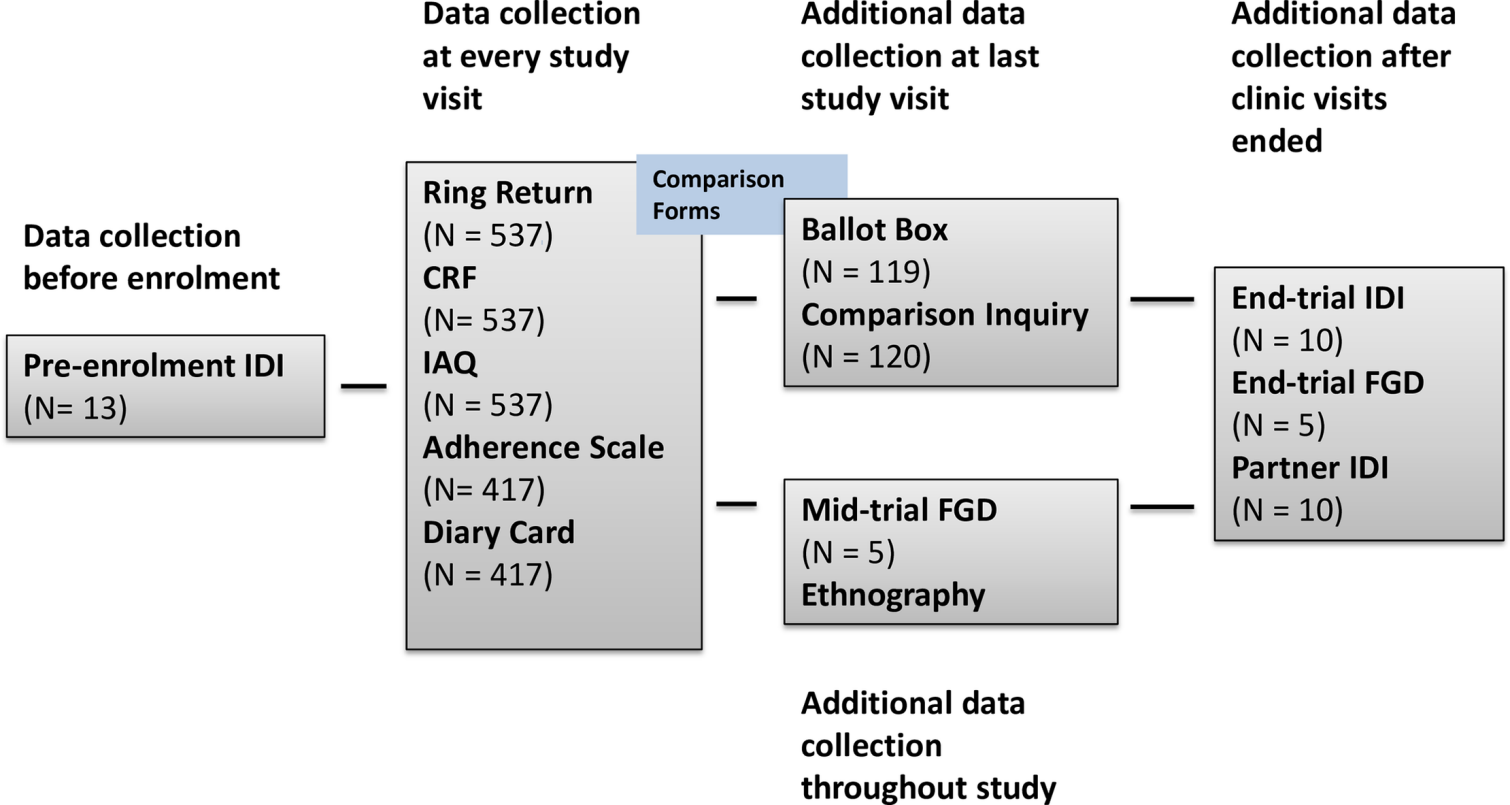
Flow chart of quantitative and qualitative data collection in the Ring Plus study. Quantitative and qualitative data collected before, during and after study end are presented.

We collected quantitative ring use acceptability data as part of the CRFs and IAQs at screening, at enrolment after the first ring insertion, and at every subsequent ring removal follow-up visit [Fig 2]. In addition, at enrolment and each follow-up visit participants completed a self-rating adherence scale and received a diary card to document ring removals and expulsions as well as sexual and vaginal practices that occurred in between study visits within their home. We compared the different data sources for each participant at each data collection time point using comparison sheets to identify discrepancies between the data sources. At the last study visit, the study nurses asked participants about any discrepancies identified over the course of the study using open-ended questions that were individually tailored. Finally, we collected anonymous ballot box questionnaires, including a few questions on acceptability, for all participants at the last study visit.

We collected qualitative data at several time points before, during and after the study using IDIs and FGDs [Fig 2]. At the pre-screening visit, 13 IDIs were conducted to adapt and refine the correct meaning and understanding of the structured IAQs prior to enrolment [36–37]. Midway through the study, five mid-trial FGDs with eight to 12 women from both the intermittent and continuous groups (total of 51 women) were conducted to explore CVR acceptability in depth, and to elaborate on contextual issues such as vaginal practices and family planning. We purposively selected women based on previous family planning use (or lack thereof) as well as documented problems using the ring such as deliberate removals and/or spontaneous expulsions. Once the study was completed, we conducted five end-trial FGDs with a total of 49 women who had not previously participated in a FGD and were purposively selected based on their responses to IAQs to further explore their perceptions about the ring and their experiences with deliberate removals and/or spontaneous expulsions. In addition we conducted IDIs with 10 study participants and 10 male partners (not necessarily linked to each other). We purposively selected those 20 interviewees based on specific study related data pertaining to ring acceptability. All IDIs and FGDs were based on structured list of questions and conducted by the same (female) moderator. A male moderator conducted the male partner IDIs. We trained the moderators and allowed them to probe if deemed necessary.

### Data analysis

We analyzed the quantitative data using STATA (StataCorp. 2013. Stata Statistical Software: Release 13. College Station, TX: StataCorp LP.) and reported the results in contingency tables (frequency for categorical data; median and interquartile range (IQR) for continuous data). The authors combined the acceptability data of the two clinical trial groups in this paper because we did not find any statistically significant differences between both groups in those areas.

**Fig 3:**
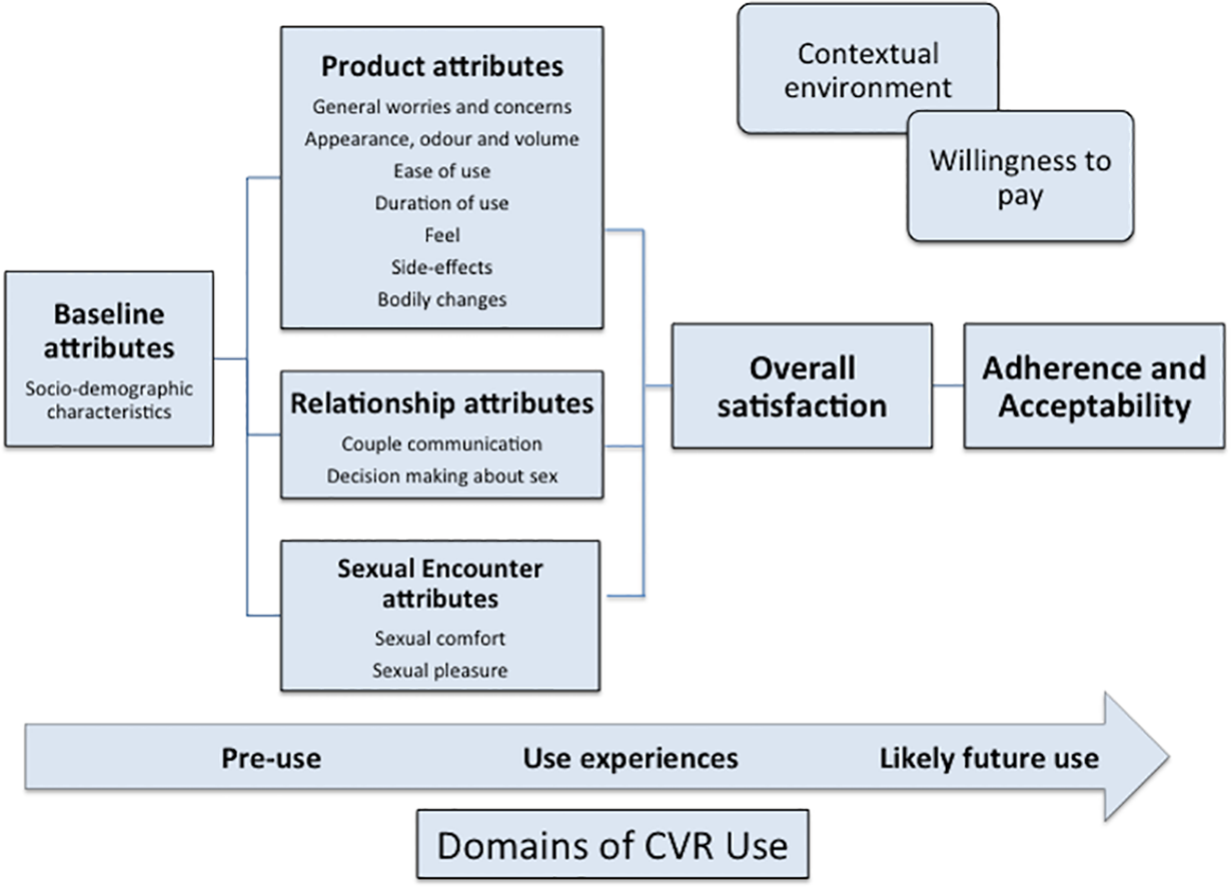
Theoretical framework used for data collection and analysis as adapted from Van der Straten et al [38] and Merkatz et al [40]. The framework proposed by van der Straten et al [35] was used for data collection and analysis; elements of the NES/EE CVR acceptability model by Merkatz et al [37] such as side effect and bodily changes were added.

The study team audio-recorded, transcribed verbatim, and translated all IDIs and FGDs into English. The English transcripts were uploaded into Nvivo 10. We analyzed this data using a deductive, content-analytical approach to assess if the components of the holistic theoretical framework developed by van der Straten (2012) for assessing acceptability of vaginal rings for microbicide delivery were valid in our setting with CVR use [38–39]. The framework includes baseline attributes (socio-demographic attributes), followed by product attributes, relationship attributes, sexual encounter attributes, and the contextual environment. Components from Merkatz’s model on CVR acceptability (2014), such as side effects and satisfaction, were integrated into van der Straten’s framework as well as new elements that emerged from the data [Fig 3] [40]. Two qualitative researchers performed all coding and analysis to compare their coding and reach consensus on divergent issues.

## Results

We collected quantitative data for the 120 women enrolled in the trial. One participant withdrew consent after enrolment and did not complete any of the scheduled study visit CRFs or IAQs but she did complete an end of study visit. In total, 104 of the 120 enrolled women participated in at least one IDI and/or FGD and 8 participants had both an IDI and a FGD. The remaining 16 were either not available to attend or not selected. Results are presented based on the components of the van der Straten framework [38–39].

### Baseline characteristics of the study population

Overall, the socio-demographic characteristics between the two clinical trial groups (intermittent and continuous users) were very similar [Table 1]. The median age of the enrolled women was 28 (IQR: 26–31.9) and the median age of first intercourse was 18 (IQR: 16.5–18.5). The majority of the women had primary school level of education and were married (60.8%). Regarding contraception, 65.8% of the women had ever used a contraceptive method (besides condoms) and most felt that the best family planning method was either injectables (30%) or condoms (30.8%). Most participants (48.3%) reported using condoms sometimes but not always in the past three weeks.

**Table 1:** Socio-demographic characteristics of enrolled participants.

Baseline characteristics, N (%)	Intermittent use	Continuous use	All participants
	(N = 60)	(N = 60)	(N = 120)
**Age in years (median; IQR)**	28 (25.5; 31)	28.5 (26; 32)	28 (26; 31.9)
**Highest Level of Education**			
No schooling	9 (15.0)	6 (10.0)	15 (12.5)
Primary school not completed	15 (25.0)	21 (35.0)	36 (30.0)
Primary school completed	24 (40.0)	20 (33.3)	44 (36.7)
Secondary school not completed	8 (13.3)	9 (15.0)	17 (14.2)
Secondary school completed	2 (3.3)	2 (3.3)	4 (3.3)
More than secondary school	2 (3.3)	2 (3.3)	4 (3.3)
**Marital Status/Home Situation**			
Married	37 (61.7)	36 (60.0)	73 (60.8)
Not married, regular sex partner, living together	16 (26.7)	16 (26.7)	32 (26.7)
Not married, regular sex partner but not living together	7 (11.7)	8 (13.3)	15 (12.5)
**Income**, Rwandese Franc (RwF)			
Own Income	37 (30.8)	34 (28.3)	71 (59.2)
Average weekly Income	16.828 RwF	24.413 RwF	20.685 RwF
**Age of first intercourse, in years (median, IQR)**	18 (16–18)	18.5 (16.5–20)	18 (16.5–18.5)
**Lifetime male sexual partners:**			
1–3	51 (85.0)	53 (88.3)	104 (86.7)
4 or more (range 4–300)	9 (15.0)	7 (11.7)	16 (13.3)
**Other sexual partners in past 3 months**	2 (3.3)	2 (3.3)	4 (3.3)
**Pregnancies**			
0	2 (3.3)	3 (5.0)	5 (4.2)
1	12 (20.0)	10 (16.7)	22 (18.3)
2	22 (36.7)	17 (28.3)	39 (32.5)
3 or more	24 (40.0)	30 (50.0)	54 (45.0)
**Contraception history**			
None	19 (31.6)	22 (36.6)	41 (34.2)
Contraceptive	41 (68.3)	38 (63.3)	79 (65.8)
Injectables^†^	32 (53.3)	27 (45.0)	59 (49.2)
Pills^†^	11 (18.3)	18 (30.0)	29 (24.2)
Implant^†^	2 (3.3)	3 (5.0)	5 (4.2)
IUD (Copper)^†^	1 (1.7)	0 (0)	1 (0.8)
Beads or counting^†^	2 (3.3)	0 (0)	2 (1.7)
Best FP in your opinion*			
Condoms	15 (25.0)	22 (36.7)	37 (30.8)
Injections	19 (31.7)	17 (28.3)	36 (30.0)
Oral contraception	11 (18.3)	7 (11.7)	18 (15.0)
Implant	6 (10.0)	4 (6.7)	10 (8.3)
IUD (Copper)	4 (6.7)	4 (6.7)	8 (6.7)
Beads and Counting	2 (3.3)	2 (3.3)	4 (3.4)
Condom use**			
Always	12 (20.0)	9 (15.0)	21 (17.5)
Sometimes but not always***	24 (40.0)	35 (58.3)	59 (49.2)
Never	23 (38.3)	16 (26.7)	39 (32.5)

† More than one answer possible

* Question asked as ‘What is the best method for family planning, in your opinion?’

** Defined as condom use during vaginal sex in the past three weeks.

*** This was the only characteristic that was significantly different between the two arms (p = 0.045).

### Product attributes

#### General worries and concerns

We asked women at the enrollment visit, prior to initiation of ring use, what worries they had, if any, about using a CVR. Overall, 31.7% of the women had at least one worry, with the top four worries noted as the ring might come out (10.8%), be uncomfortable during sex (9.2%), cause infection (7.5%), or not adequately protect against pregnancy (7.5%). The worries about ring expulsions were echoed during IDIs and FGDs conducted after the first ring insertion:

*“When I inserted the ring for the first time, I was worried, every time I had to go to the toilet, I would feel like it [the ring] would come out, I had to protect it using my hands…so that if the ring came out, it would fall into my hands. But this never happened.” (*27, unmarried, 1 child*)*

During the IAQs at ring removal visits, 16% of the women reported at least one worry about ring use at least one time during the study. However, at the last ring removal visit, only four women still reported worries with two women reporting that they were worried about their partner not liking the ring and two women reporting to still be worried about ring expulsions.

#### Appearance, odor, and volume

According to the IDI and FGD data, the majority of the women had no issues with the appearance, odor, and volume of the ring. A few women noted that they were concerned with the size before they had ever inserted a ring, while others argued that if it were any smaller, it might not fit all vaginas. When asked to describe the ring, women described it as soft and cold with either no smell, smelling like chewing gum, or a pleasant smell. They also noted that the ring was white or beige when inserted but came out yellow after use. The women were, however, not concerned about this because they had been informed about potential discoloration during the study visits.

Male partners of study participants voiced several concerns about the ring during IDIs (n = 10) at the end of the study. Most of the concerns were related to potential side effects of ring use (e.g. illness, infertility) and about ring appearance. Male partners did not have an opportunity to see the ring prior to insertion, and they associated the word ‘ring’ (in Kinyarwanda *impeta*) with a metal ring. They were reassured when they eventually saw a ring.

*“I wanted to see what it looked like, when I saw it, I found out that it is flexible, it would not cause cancer, nothing on it that would harm people, so I told her, you insert it, I am not worried about it and I did not experience any problem.” (*30, married, 2 children*)*

#### Ease of use

The quantitative and qualitative data revealed that, overall, women found the ring easy to insert and remove. At the enrolment visit, immediately after inserting the ring for the first time, only five participants said that they did not find the ring easy to insert and two participants said that insertion was painful. Almost all women (96.7%) thought that reinsertion would be easy. By the end of the study, all but one participant stated that ring insertion was easy.

#### Duration of use

During FGDs and IDIs most participants thought that the duration of use of each individual ring should be increased (i.e. longer than the current 3 weeks) with suggested preferred use durations ranging from 2 months to 10 years. Women compared these durations to other methods “*like 3 months injection*, *like 3 years implant*, *like 10 years IUD*”. Reasons cited for preferring increased use duration were potential lower costs, having to remove and insert the ring less often, and maintaining increased vaginal lubrication as women reported that the ring increased vaginal lubrication but that “*the lubrication went down when the medicine [in the ring] finishes*”. Most male partners tended to have questions about the duration of ring use (e.g. “*how long should it be used*?”) and one partner said the duration was too short.

#### Feel

Most women liked that they did not feel the ring during daily activities (reported by IAQ by 95.4% of the women at ring removal visits during ring use and by 95.8% of the women at the last ring removal visit) but a few women said that not feeling the ring made them worry that it had fallen out. During interviews only very few participants reported that the ring caused some discomfort during use, and one of those women reported that the initial discomfort was caused by improper insertion.

#### Bodily changes

Women reported an overall increase in vaginal wetness or lubrication in the IAQs (52.9% of the women at least once during ring use and 74.8% at the last ring removal visit). This was generally perceived as a positive attribute, which was corroborated by the qualitative data. Only two women reported that they did mind the increased lubrication but in their opinion, their partners did not mind it. At the last ring removal visit, one of those women still reported the increased lubrication was a problem for her but not for her partner.

At the last ring removal visit, 8.3% of the intermittent users and 57.6% of the continuous users reported to no longer have menstrual periods. This was not perceived as a problem except for one continuous user:

*“I feared that I got a tumor in my uterus because my menses stayed inside my body I am wondering where the menses goes if they are not coming out?”(32*, *unmarried, 1 child)*

#### Side effects

Women appreciated the lack of negative side effects such as headache and back pain, which they perceived as common side effects of other hormonal contraceptive methods.

*“I stopped using the other methods because of those side effects that used to happen to me. I even had Norplant, but stopped it because of dizziness, headache, bleeding, all those were going to kill me. But since I have started using this ring, nothing happened to me”. (*35, married, 6 children)

### Relationship attributes

In the IAQs, we asked women how they felt about their relationships in general. At the last ring removal visit, 60.8% of the women reported to be very satisfied, and 37.5% moderately satisfied, in their relationships. Only one woman reported to be very dissatisfied in her relationship. 60% of participants described the most important factors for a happy and satisfactory relationship as having a partner who helped, cared for and/or provided (financially) for the family. Women also found sharing responsibilities and discussing or planning together important (25.8%) for a satisfactory relationship whereas alcohol abuse (11.7%) and having other partners (6.7%) were the main causes of dissatisfaction. Two women reported cases of physical abuse (including one rape).

#### Couple communication

At enrolment 93.3% of the women had told their main partner that they would be wearing the ring and the eight women who had not yet told their partners but intended to do so later on. At the last ring removal visit, all but one woman had told their partners that they were participating in a vaginal ring study. These attitudes about contraceptive use and communication within the couple were echoed in interviews as well:

“*If the man does not want you to use contraceptives, then you have to accept what he asks you to do, he is the head of the house”. (25, not married, 1 child)*

#### Decision making about sex

Based on the qualitative data collected, it was clear that women in our study felt that if they are not satisfied during sex, they cannot tell their partners. They thought that if a woman does not want sex, the man would force himself onto her. However, they also reported that women had ways to get around this, for example, by pretending to have menses.

An interesting finding was that most women reported that ring use stimulated conversations with their partners about increased lubrication and sexual desire, but also about family planning and more general relationship topics. Mutual sexual gratification is seen as an important element in the relationship and women felt that if sex is mutually satisfying, overall couple communication is better.

### Sexual encounter attributes

#### Sexual comfort

At the beginning of the study, before ring use, 58.3% of the women thought it important that their main partner would not feel the ring during sex. At the last ring removal visit, 82.5% of the women reported to never have felt the ring during vaginal sex. However, 52.5% of the women reported that their partner had told them that he had felt the ring during vaginal sex at least once during ring use. Before first ring use, women thought that whether or not their partner actually felt the ring would not affect acceptability but if the ring caused either of them discomfort during sex, acceptability would be negatively affected. Once women had experienced using the ring, only 3.3% reported at least once during ring use that their partner disliked the way it felt during sex. At the last ring removal visit, no woman reported that her partner disliked the way it felt during sex. This was corroborated by the IDIs with male partners: six of the 10 interviewees reported having felt the ring during sex but said that this was not a problem.

#### Sexual pleasure

Most women (80.6%) reported at least once during ring use that the ring made sex feel better and at the last ring removal visit, this increased to 87.5%. The FGDs and the IDIs confirmed this finding [Fig 3].

*“Really I would not feel any desire of men. It is true, when I was using contraceptive injections I was like a man*. *I was very dry. But today, I am the one who calls him and tells him that I am feeling well and actually tells him that I am ready for sexual intercourse.” (31, married, 5 children)*

The increased vaginal lubrication as well as increased sexual desire led one participant’s partner to state, “*This ring should be promoted as a sex enhancer*”.

### Overall satisfaction

Overall satisfaction has been shown to be a good indicator of future use and adherence to a product. In our study we found that women were very satisfied with only one woman at enrollment stating that she thought that the CVR was the best method for family planning compared to 98.3% of the women stating this at the last ring removal visit. These same women also said that they would recommend a CVR to others. This is in the context of only two women ever having used a vaginal ring prior to study participation.

### Willingness to pay

The cost of a family planning method needs to be taken into consideration when evaluating overall acceptability. We asked how much women would be willing to pay for a CVR like the one they wore in the study. Only 0.3% women stated that they would not be willing to pay for the ring but that they would prefer it to be available free of charge at family planning centers. Not a single woman reported that she was willing to pay more than 17,000 Rwandan francs (RwF) per ring (equivalent to about 20 USD at the time of writing), and most women (95.8%) reported to be willing to pay between 1–8,500 RwF (up to 10 USD) per ring.

### Contextual environment

The contextual environment defined here as the overall beliefs and attitudes about family planning and sexuality in Rwanda, the community perceptions of research, and the experiences of participation in this trial, plays a large role in the overall acceptability and uptake of any new product. Many participants and their partners were aware of the fact that the Rwandan government recommends three children per family and child spacing. This was apparent in the FGDs, in which women mentioned that a typical family has two to four children, and cited the benefits of spacing children.

Community perceptions about research relayed by enrolled and excluded women and their partners seemed polarized. Screened participants mentioned rumors in all end trial FGDs and several IDIs. When probed about these rumors, women elaborated about their own communities and experiences mentioning as the main rumor that at the study site (Rinda Ubuzima), the uterus and ovaries were removed and sold or thrown out. Community members were also worried about sterilization due to organ removal, to ring use, or to both. Enrolled participants reported that other women often did not join the study because they feared the pelvic exam and the ring itself could cause health problems, such as bleeding or cancer of the area where it was placed, and even death. Despite these negative rumors, community members actively tried to be recruited into the study for a variety of reasons: access to the ring, monetary reimbursement for travel and time spent at the clinic, and testing and treatment for curable STIs free of charge.

## Discussion

Like any new product, initial worries were recorded (by 31% of the women), but reduced over time with usage; in the beginning worries were focused on spontaneous expulsions but this changed during the course of the study with worries becoming more focused on male partner perceptions and concerns. Once the participants had ring use experience, the method of contraception perceived as being the best, changed from condoms (reported by 30.8% of the women at enrollment) to NuvaRing® (reported by 98.3% of the women at the last ring removal visit). A high level of acceptability following actual use has been documented in other studies of new contraceptive or HIV prevention methods, and suggests that self-reported acceptability based on hypothetical use might not be valid [18]. Appropriate counseling regarding correct use and side-effects can reduce anxiety associated with first time use and as a result enhance initial uptake and acceptability [18, 24, 40–41].

Self-reported comfort with one’s genitals as well as experience using other vaginal products such as tampons were significantly associated with willingness to try the vaginal ring in a number of studies, but not in others [14, 42–44]. None of our participants reported to ever have used tampons, but vaginal sexual and hygiene practices are common in Rwandan culture. As a result, most women are comfortable with their genitalia [41,45–46]. Previous research has shown the important role of sexual gratification in sexual encounters for both women and men [41]. These cultural norms around sexuality, including vaginal practices such as labial elongation as well as ‘wet’ sex, positively influenced the acceptance of NuvaRing® [45–46]. The participants in our study engaged in several traditional vaginal practices and found the ring easy to use. In terms of acceptability, the ring’s appearance did not seem to be an important issue, in line with findings from a progesterone-only CVR study in three African countries [20], but in contrast to findings from a progesterone-only CVR study in Brazil [22]. The latter showed that 32% of the participants did not like the discoloration of the ring, referring to it as dirty [22].

The lack of side effects was seen as a major advantage over other family planning methods as was the increased lubrication. This is in line with Rwandan cultural sexual norms where both men and women see ‘wet’ sexual intercourse as desirable. These results highlight the importance of the contextual elements. Further research is needed in other countries that have different cultural norms around sexuality such as settings where dry sex is a common preference. Most participants would have preferred to leave each individual ring in place for longer than the currently licensed three weeks, as has been shown in other studies [26–27]. As stated above, the fact of no longer having of menstrual periods was not perceived as a problem in our study.

Overall, no women reported that using the ring had negative effects on their sexual relationships and feeling the ring during intercourse was not an important issue. Prior to using the ring, women felt that discomfort for their male partner during sex related to the ring would impact acceptability but after using the ring, the majority of women did not report that the ring caused their partner discomfort. These findings regarding sexual comfort are in line with other studies [18–19]. Disturbances of sexual intercourse for a variety of reasons are crucial determinants of acceptability and adherence as well as good indicators of method continuation as noted by Sabatini [47]. However, the literature is not consistent regarding the effect of ring use on sex frequency and pleasure. Some studies have shown no effect on sex frequency and only a slight effect on pleasure but other studies, like our study, have shown a significant improvement in pleasure accompanied by increases in sex frequency [24, 48].

The CVR is a female initiated product but women felt that their partners needed to know that they were using the ring (even if they did not feel it during sex) since the man, as head of the household is seen as the decision maker regarding family planning. This is in line with previous studies highlighting that relationship attributes play a significant role in assessing overall acceptability (Woodsong et al 2008) [39]. Being part of the study and using the ring led to more communication within the couple about their sexual relationships and about family planning. This in turn led to more discussions surrounding decision-making about sex. Most women felt that this increased communication improved their overall relationship. This interesting finding has been noted in other studies and could be a potential avenue for increased family planning counseling/couple counseling during and after clinical trial implementation [48].

## Limitations

Our study included a relatively small number (N = 120) of women willing to participate in a clinical trial, at high risk of HIV and other urogenital infections and from an urban population in Kigali. Such attitudes and beliefs towards CVRs might be different in rural areas of Rwanda and are not generalizable to the whole country. In addition we did not have a control group of women who used other modern family planning methods or used none at all. A substantial proportion of the study participants had, however, used other modern family planning methods prior to joining our study. They could therefore compare their experiences with CVR use to other methods.

It is well documented that data on acceptability and acceptability within a clinical trial setting do not necessarily reflect the real-life situation due to higher participant and study staff motivation, higher levels of support provided by study staff, and pro-active follow-up [49, 50]. When rolling out a new product to a larger population, product packaging, labeling, storage requirements, and service delivery should also be assessed. We did not do so but note that NuvaRing can be stored at room temperature [39, 51]. Should NuvaRing be seriously considered for roll out in Rwanda, it would also be important to assess the views of policy makers and advocacy groups [51].

## Conclusions

Acceptability of the Nuvaring was high in Kigali, Rwanda. Rolling out this method would increase the contraceptive method mix and lower unmet family planning need. The CVR is a female initiated method but, through the analysis of both quantitative and qualitative data, couple communication and involvement of the male partners in contraception/family planning decision-making appeared crucial in acceptance of the method. Although we did not assign values to the attributes, our data suggest that ring acceptability depended more on the relationship and socio-cultural context than on product attributes. We therefore believe that it is important that acceptability studies take a holistic approach by taking not only contraceptive efficacy and side effects into account but also the broader potential benefits including effects on sexuality.
